# Female Preference for Sympatric vs. Allopatric Male Throat Color Morphs in the Mesquite Lizard (*Sceloporus grammicus*) Species Complex

**DOI:** 10.1371/journal.pone.0093197

**Published:** 2014-04-09

**Authors:** Elizabeth Bastiaans, Mary Jane Bastiaans, Gen Morinaga, José Gamaliel Castañeda Gaytán, Jonathon C. Marshall, Brendan Bane, Fausto Méndez de la Cruz, Barry Sinervo

**Affiliations:** 1 Department of Ecology and Evolutionary Biology, University of California Santa Cruz, Santa Cruz, California, United States of America; 2 Nanooptical Materials, Incorporated, Carson, California, United States of America; 3 Facultad en Ciencias Biológicas, Universidad Juárez del Estado de Durango, Gómez Palacio, Durango, México; 4 Department of Zoology, Weber State University, Ogden, Utah, United States of America; 5 Laboratorio de Herpetología, Departamento de Zoología, Instituto de Biología, Universidad Nacional Autónoma de México, Distrito Federal, México; Columbia University, United States of America

## Abstract

Color polymorphic sexual signals are often associated with alternative reproductive behaviors within populations, and the number, frequency, or type of morphs present often vary among populations. When these differences lead to assortative mating by population, the study of such polymorphic taxa may shed light on speciation mechanisms. We studied two populations of a lizard with polymorphic throat color, an important sexual signal. Males in one population exhibit orange, yellow, or blue throats; whereas males in the other exhibit orange, yellow, or white throats. We assessed female behavior when choosing between allopatric and sympatric males. We asked whether females discriminated more when the allopatric male was of an unfamiliar morph than when the allopatric male was similar in coloration to the sympatric male. We found that female rejection of allopatric males relative to sympatric males was more pronounced when males in a pair were more different in throat color. Our findings may help illuminate how behavioral responses to color morph differences between populations with polymorphic sexual signals contribute to reproductive isolation.

## Introduction

Discrete phenotypic polymorphisms in colorful sexual signals are associated with alternative reproductive tactics in a variety of taxa [1]–[9], including several lizards [3], [10], [11]. Taxa in which color polymorphisms or alternative reproductive tactics are common may diversify at higher rates than taxa that do not exhibit these characteristics [12]–[17], a hypothesis supported by the observation that closely related species or divergent populations of the same species often differ in their sexual signals [18], [19]. These differences may initially arise in allopatry [20]–[23], in parapatry [24], [25], after secondary contact [26]–[28], or in sympatry [29]–31. Regardless of which evolutionary mechanism gave rise to divergent sexual signals in a particular system, there are numerous cases in which individuals mate preferentially with members of their own populations based on sexual signals that differ between populations or closely related species [31]–[37].

In several lizards in which color morphs vary in behavior within populations, there is also variation among populations in morph frequencies, or in which combinations of color morphs are present in particular populations. This pattern may represent an intermediate stage in the transition between polymorphic species in which all populations are linked by gene flow and dimorphic or monomorphic species which are reproductively isolated [16], [22], [38], [39]. A recent study assessed reproductive isolation between two parapatric populations of the color polymorphic lizard *Uta stansburiana* that differed in habitat, dorsal coloration, and number of throat color morphs present. Color morphs varied in the degree of postzygotic reproductive isolation they displayed in interpopulation crosses, indicating that which color morphs are present, and the frequencies at which they occur, may influence the degree of gene flow between color polymorphic populations [40].

Polymorphic coloration in lizards may be important in intrasexual signaling among both males and females [1], [3], [41]–[43] as well as intersexual signaling and/or mate choice [2], [44]–[48]. Lizards in which sexual color signals vary at both the intra- and interpopulation levels may therefore be ideal systems for understanding the importance of divergence in sexually selected traits before, during, and after speciation [14], [15], [19], [24].

The mesquite lizard (*Sceloporus grammicus*) species complex is an excellent system in which to investigate intrapopulation color and behavior polymorphisms, variation among populations in sexual signals, and the roles both kinds of variation may play in speciation. The species complex occurs in desert and montane habitats in Mexico. It consists of eight chromosome races, among which the diploid number varies from 32 to 46 [49]–[51]. This karyotypic variation may indicate that the *S. grammicus* complex is an example of incipient speciation [49], [51]–[53]. Several hybrid zones between different chromosomal races exist [54]–[58], with reduced hybrid fitness occurring in at least one case [59]. Populations of the *S. grammicus* complex also vary in life history, habitat use, and morphology [53], [60]–[64].

**Figure 1:**
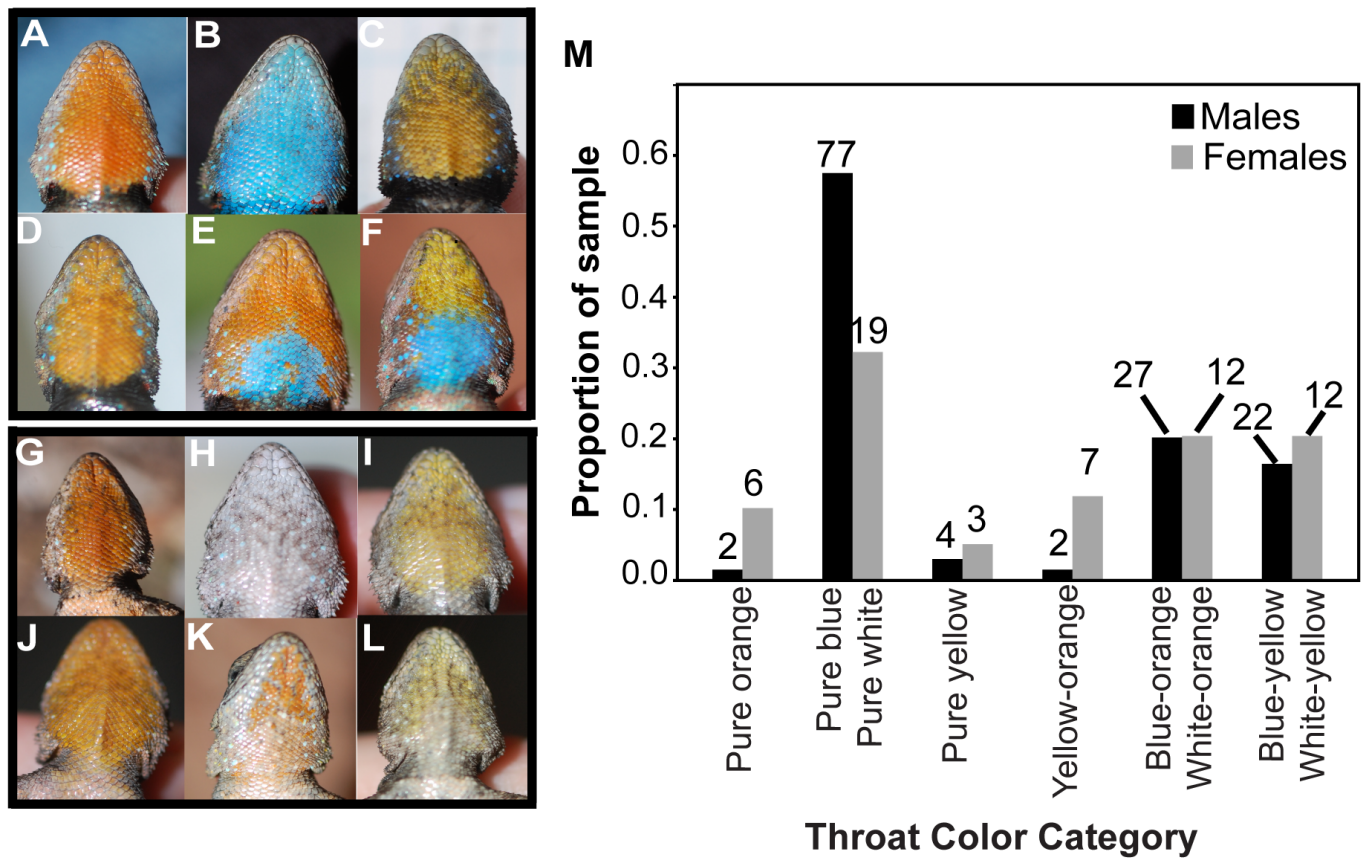
Male and female throat color morphs present in *S. grammicus* at CPN. A–F: Male morphs. A. Pure orange. B. Pure blue. C. Pure yellow. D. Yellow-orange. E. Blue-orange. F. Blue-yellow. G–L: Female morphs. G. Pure orange. H. Pure white. I. Pure yellow. J. Yellow-orange. K. White-orange. L. White-yellow. M. Frequencies of male and female throat color morphs among all lizards captured at CPN in 2011. Sample sizes above bars.

**Figure 2:**
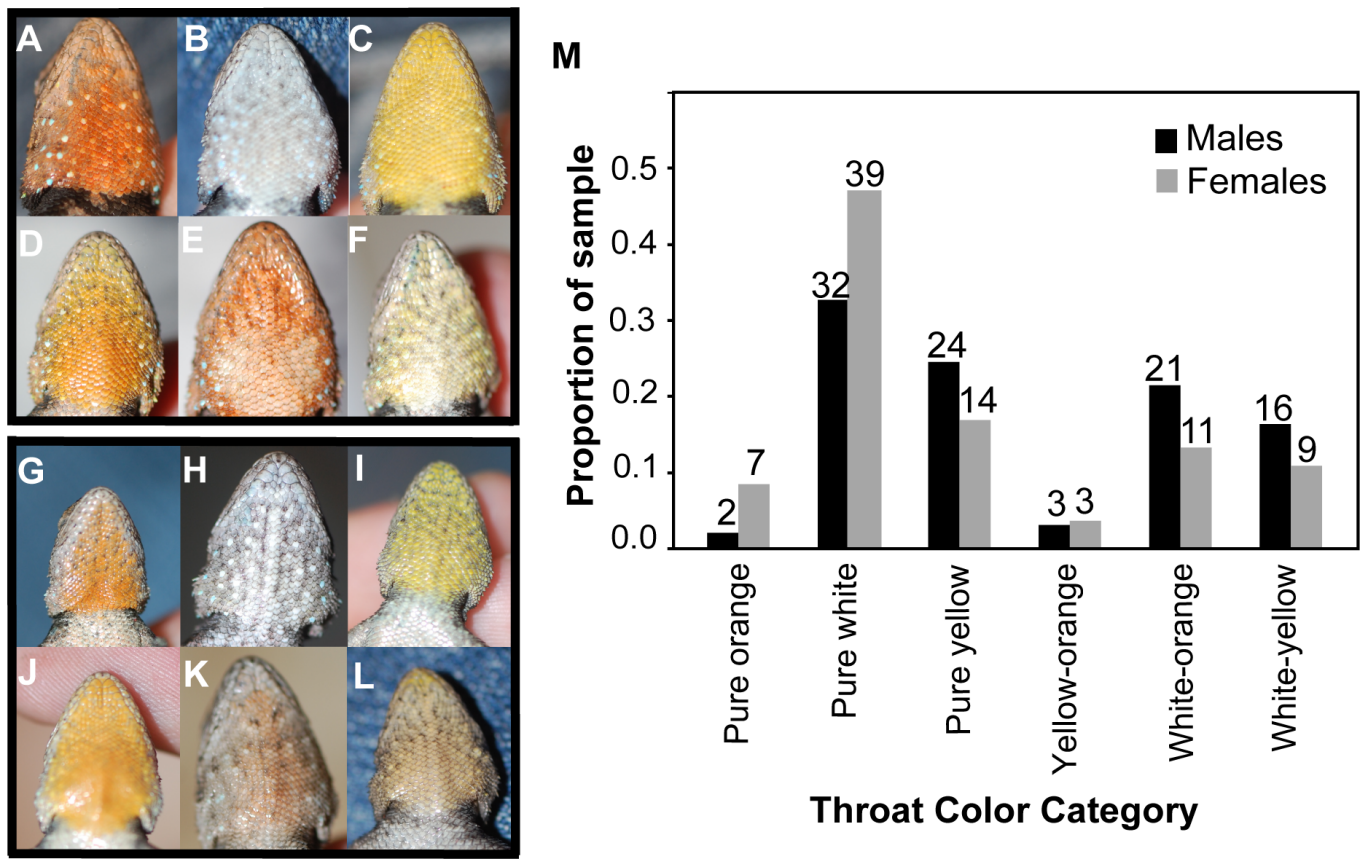
Male and female throat color morphs present in *S. grammicus* at SAA. A–F: Male morphs. A. Pure orange. B. Pure White. C. Pure yellow. D. Yellow-orange. E. White-orange. F. White-yellow. G–L: Female morphs. G. Pure orange. H. Pure white. I. Pure yellow. J. Yellow-orange. K. White-orange. L. White-yellow. M. Frequencies of male and female throat color morphs among all lizards captured at SAA in 2011. Sample sizes above bars.

Intrapopulation throat color polymorphisms occur in both males and females of the *S. grammicus* species complex [65]. In some populations, males exhibit orange/yellow/blue color polymorphisms (Fig. 1A–F) that are phenotypically similar to the polymorphisms described in two confamilial lizards, *Uta stansburiana* [3] and *Urosaurus ornatus* [66]. In other populations, males exhibit orange/yellow/white throat color polymorphisms (Fig. 2A–F) that appear more similar to the throat color polymorphism in the congeneric *Sceloporus consobrinus* (formerly *Sceloporus undulatus erythrocheilus*) [67], [68]. Blue-throated and white-throated male *S. grammicus* have never been observed within the same population [69]. Female *S. grammicus* in both types of populations exhibit orange/yellow/white throat color polymorphisms (Fig. 1G–L, Fig. 2G–L).

The presence of blue-throated males in some populations and white-throated males in others is the most striking feature of interpopulation sexual signal variation in the *S. grammicus* species complex, and there are cases in which populations with blue males occur in close proximity to populations with white males but retain their morph differences [69]. The circumstances under which the divergence in throat color originally occurred are not known. If the divergence in sexual signals initially occurred in sympatry or parapatry, or if the divergence began in allopatry but was completed in parapatry, blue vs. white variation might represent reproductive character displacement due to reinforcement [26], [70]. However, this hypothesis does not explain why the yellow and orange male morphs have not diverged. If the substitution of one morph for another was completed in allopatry, it may have occurred because some environments favor blue-throated males, whereas other environments favor white-throated males [19], [23], [30], [71]. Alternatively, divergent runaway processes in allopatry may have culminated in the presence of white-throated males in some populations but blue-throated males in others [12], [19], [72]. Under any of the above scenarios for the origin of the blue and white male morphs, females might be less likely to accept males as mates if those males exhibit a throat color that does not occur in the female’s home population. This prediction holds if females are reproductively incompatible with allopatric males and does not necessarily depend on how that incompatibility arose [6], [9], [36], [40], [72]–[73]. Our purpose in this study is not to distinguish between alternative hypotheses for how the blue and white male morphs initially arose, but rather to assess whether they currently have effects that could help explain patterns of gene flow within the *S. grammicus* species complex.

If females respond to male throat color, we expect females from populations with blue males to readily identify white-throated males as allopatric, whereas females from populations with white males readily identify blue-throated males as allopatric. When choosing between one sympatric and one allopatric male, we expect females to discriminate more when the allopatric male is of an unfamiliar morph, compared with choices between males of the same color morph. We tested this hypothesis using a repeated-measures, binary choice design. We predicted that individual females would reject allopatric males more strongly when choosing between allopatric and sympatric males differing completely in throat color than when choosing between allopatric and sympatric males that were more similar to one another in throat color.

## Materials and Methods

### Ethics Statement

This project was conducted in strict accordance with guidelines from the Institutional Animal Care and Use Committee (Chancellor’s Animal Research Committee) at the University of California, Santa Cruz (permit Sineb0902). Lizards were collected under permits issued by the Secretaría del Medio Ambiente y Recursos Naturales of México (folio FAUT007). When sampling took place on private land, we obtained permission from property owners prior to collecting animals.

### Field Sites

We used lizards from two field sites: Cerro Peña Nevada, Nuevo León (23.83154°N, 99.89381°W; 2800 m above sea level) and San Antonio de las Alazanas, Coahuila (25.22193°N, 100.39331°W; 2800 m above sea level). For brevity, we will refer to these two localities as CPN and SAA, respectively. Lizard habitat was similar at both sites and consisted primarily of conifers such as *Pinus, Abies,* and *Pseudotsuga spp.* [75], [76]. Additional details regarding these two field sites are provided in [65]. Male lizards at CPN exhibit an orange/yellow/blue throat color polymorphism (Fig. 1), whereas males at SAA exhibit an orange/yellow/white polymorphism (Fig. 2). The two localities are approximately 180 km apart [77], so there is probably no direct migration between them. However, both sites are located within the Sierra Madre Oriental mountain range, throughout which *S. grammicus* is common at high elevations [50], [78], [79], so we cannot rule out the possibility that CPN and SAA are linked by an unbroken chain of other populations. *Sceloporus grammicus* from these two localities are closely related, based on allozyme electrophoresis studies, and the two populations likely both belong to the same chromosomal race (2N = 32) [50], [79], which indicates they might not exhibit reduced hybrid fitness, although no tests of this hypothesis have been performed. Mitochondrial and nuclear DNA-based phylogeographic studies also support a close relationship between CPN and SAA [69].

### Lizard Capture and Husbandry

From July-September, 2011, we captured 59 adult females from CPN (Fig. 1M) and 83 adult females from SAA (Fig. 2M). Due to the constraints of our experimental design (see below), we used only 17 randomly-chosen adult females from each population in our mate choice experiments [46]. Similarly, we captured 134 adult males from CPN (Fig. 1M) and 98 adult males from SAA (Fig. 2M) but used only 46 males from CPN and 35 males from SAA in our experiments. We captured all lizards by hand or by noose. We considered lizards adults if they had secondary sexual coloration (blue belly patches in males and throat patches in both sexes) and a snout-vent length (SVL) greater than 40 mm [80], [81].

Within one week of capture, we transported lizards to the Universidad Juárez del Estado de Durango (UJED), Gómez Palacio, Durango, México. We isolated males and females in separate coolers during transport and kept individual lizards in cloth bags to prevent interactions between them. At UJED, we maintained all lizards in a room with large windows open to the ambient air and UV light. Lizards were kept in individual terraria with opaque walls. We misted terraria with water daily and fed the lizards 4–5 crickets (*Acheta domesticus*) every other day. We placed the terraria on Flexwatt © heat tape, coupled to a ZooMed thermostat set at 32°C [82]. We allowed lizards to acclimate to the laboratory for at least 5 days before we used them in behavior trials [83]. At the end of our study (October 2011), we released all lizards at their original capture locations.

### Throat Color Scores

We scored throat color using methods developed for *Uta stansburiana*, a confamilial lizard with a phenotypically similar throat color polymorphism. In *U. stansburiana*, the throat color polymorphism is genetically based and appears to be controlled by either one locus with orange, yellow, and blue alleles or two very tightly linked loci, based on breeding studies [3], [16], [46], [84]–[87] and gene mapping [88]. For details regarding how we assigned color scores and assessed their repeatability, please see the Supplementary Materials (Text S1) and [65].

### Experimental Design

**Figure 3:**
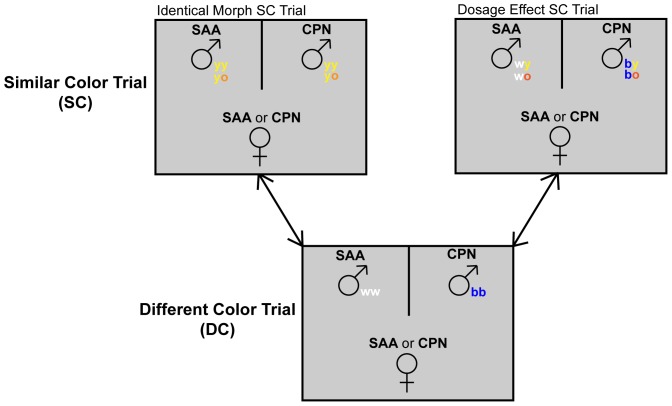
Schematic representation of our mate choice experiment. All females participated in one SC trial and one DC trial, each of which included two males, one from SAA and one from CPN. In all cases, DC trials included a pure blue male from CPN and a pure white male from SAA. In trial pairings with an identical morph SC trial, the males included in the SC trial were either both pure yellow or both yellow-orange. In trial pairings with a dosage effect SC trial, the possible male pairings in the SC trial were blue-orange (CPN) vs. white-orange (SAA) or blue-yellow (CPN) vs. white-yellow (SAA).

Each individual female participated in two mate choice trials, one designated the “different color” (DC) trial and the other designated the “similar color” (SC) trial (Fig. 3). We assigned DC and SC male pairs to each female randomly, and we randomized the order in which we performed the trials to remove any effect of the order in which females were presented with the two pairs of males [89]. We made minor alterations to the randomized trial order on an ad-hoc basis, to avoid using individual lizards in more than one trial per day. We conducted mate choice trials between 10 a.m. and 4 p.m., because those were the hours during which we g003primarily observed lizard activity in the field (E. Bastiaans & G. Morinaga, pers. obs.).

In all mate choice trials, females chose between one male from CPN and one male from SAA (i.e., one sympatric and one allopatric male). In DC trials (Fig. 3), the male from CPN was of the pure blue morph (Fig. 1B), and the male from SAA was of the pure white morph (Fig. 2B). We performed two categories of SC trials (although any given female participated in only one), “identical morph” trials and “dosage effect” trials (Fig. 3). In the identical morph SC trials, the CPN and SAA males were of the same morph, which meant they could either be two pure yellow males (Figs. 1C, 2C) or two yellow-orange males (Figs. 1D, 2D).

The dosage effect SC trials were intended to assess whether females respond to intermediate morph males in which only one color patch is of an unfamiliar color in the same way they respond to males whose entire throat is of an unfamiliar color. In dosage effect SC trials (Fig. 3), we therefore used males that had one color patch in common and one color patch that differed between them. Thus, pairs could consist either of a blue-orange male from CPN (Fig. 1E) and a white-orange male from SAA (Fig. 2E); or of a blue-yellow male from CPN (Fig. 1F) and a white-yellow male from SAA (Fig. 2F).

We formed male pairs of each category randomly, under the constraint that males within a pair could not differ by more than 2 mm in SVL. We paired males of similar SVL to avoid possible confounding effects of male body size on female preference [90], [91]. However, these color morph and SVL constraints on male pair formation limited the number of possible male pairs, so that we had to choose between either using individual males multiple times in our experiments or performing a very small number of total trials. We chose to use individual males multiple times and account for the potential effects of male identity on female choice by including male identity as a random factor in our statistical analyses (see below) [36], [45].

We assigned SC and DC male pairs to each female randomly, under the constraint that the female was not captured within 40 m of either the SC or the DC sympatric male. This distance cutoff was double our previous estimate of the average diameter of male territories at CPN and SAA [65], and was intended to prevent trials from including males and females who were familiar with one another [74], [92].

### Mate Choice Trials

We conducted mate choice trials in a chamber measuring 70 cm wide x 46 cm long x 46 cm tall, with a plywood floor and Plexiglas © walls covered in opaque paper. The floor of the chamber was covered in sand from the UJED campus, and we changed the sand after every trial to prevent scent cues from influencing future trials [91], [93]. An opaque cardboard barrier extended perpendicular to the two long walls, halfway across the chamber (Fig. 3). This created two male display areas, into which we placed the two males used in a trial. Males were tethered to the back of the trial chamber with black surgical thread, which was tied loosely around their hips. The tethers allowed males to reach the front of their display areas, but they could not see around the barrier. This trial design prevented direct male-male interactions from influencing female mate choice [44], [45]. Each male display area contained a rock to serve as a substrate for displays, and we scrubbed the rocks with water after every trial to remove scent cues [91], [93]. We heated the rocks to approximately 30–35°C using lamps attached to the side of the trial chamber [65], [82]. Before each trial, we placed a cardboard barrier across the center of the trial chamber, perpendicular to the barrier between the two male display areas. This barrier divided the trial chamber into three sections: the two male display areas and a larger area in front of them. We placed the female into this front section and assigned males to either the left or right display areas randomly, to avoid bias that might result from females’ inherent preference for a certain side of the trial chamber or from lateralization of the visual aspects of mate choice or aggressive behavior [94], [95]. We allowed the lizards used in each trial to acclimate to the chamber for five minutes before raising the cardboard barrier separating the female from the males. Trials lasted for 20 minutes and were videotaped using a Flip Video digital camera (Cisco Systems) mounted on a tripod above the trial chamber. During the trials, no human observer entered the lizards’ lines of sight. Females had full access to both males, but males could not leave their display areas. This design minimized the probability that males could force females to copulate and increased the likelihood that female behaviors directed toward males would be an expression of female preference for or rejection of those males [44], [45].

### Behavior Scoring

All trial videos were scored by one observer (MJB), who was blind to the populations of origin of the lizards, the color morphs of the males, and the categories of the trials [96]. Every fourth trial was independently scored by another observer (EB), and differences were resolved through discussion. If disagreements could not be easily resolved, the trial was re-scored without reference to previous scores. The observer viewed each trial three times and recorded the behaviors of one lizard per viewing. Because the camera was positioned above the trial chamber, the males’ throat colors were not visible to the observer during scoring.

We scored female behaviors (Table 1) based on literature records of female reproductive behavior in closely related lizards, and based on a pilot experiment conducted in summer 2009 (E. Bastiaans and G. Morinaga, pers. obs.). The observer tallied the number of times a female performed each behavior during each trial, recorded the time point at which each behavior was performed, and also recorded the direction of each female behavior (left male or right male). Female behaviors were recorded as directed towards a male if she looked directly at the male or moved toward the male while performing them, or if she performed the behavior while located inside a male’s display area [45].

**Table 1:** Female behaviors scored^a^ during mate choice trials.

Behavior Name	Behavior Description	Source
Copulation	Male grasps female’s shoulder with teeth, male and femalecloacal regions come into contact.	[94], [120], [121]
LateralCompression	Female compresses sides and gular region laterally. May be performedalone or in combination with push-ups, but was scored separately.	[92], [94], [122], [123]
Push-up	Entire body raised and lowered vertically, due to bending and straighteningof either front legs alone or front and hind legs simultaneously	[92], [94], [122], [123]
Bite	Female grasps some part of male’s body with teeth	[92], [94]
Lick	Female touches some part of male’s body with tongue.	Pers. obs. E. Bastiaans & M.J. Bastiaans
Touch	Female touches some part of male’s bodywith a part of her body other than the tongue.	Pers. obs. E. Bastiaans & M.J. Bastiaans
Tail Wave	Female raises entire tail and waves it vigorously back and forth	Pers. obs. E. Bastiaans & M.J. Bastiaans
Tail Vibration	Female vibrates the tip of her tail but does not raise it	Pers. obs. E. Bastiaans & M.J. Bastiaans
Approach	Female moves toward male, while looking at male	[44]
Retreat	Female moves rapidly away from male,soon after some interaction between them	[97], [124]
Substrate Taste	Female touches snout to sand	[45]

a We recorded the number of times a female performed each behavior toward each male with whom she interacted.

To test for an effect of male display on female preference, we recorded the number of head-bobs and push-ups performed by each male. Push-ups involve raising and lowering the body, using either two legs or all four legs. Head-bobs are rapid, shallower motions of the head, usually performed after the body is raised in a motion similar to that of a push-up. Head-bobs and push-ups are the most prominent courtship behaviors performed by male *Sceloporus* [97]–[99]. However, because push-ups and head-bobs can be difficult to distinguish and are usually performed together (pers. obs. E. Bastiaans and M.J. Bastiaans), we summed them as an index of the overall intensity of male display behavior during the mate choice trials [45].

We estimated association time between a female and an individual male by looking for sequences of behaviors directed towards that male. We then subtracted the first time point from the last time point for each sequence of behaviors, and summed the time periods for all sequences of behaviors directed towards each male during the trial. For instances in which a female directed only a single behavior towards a male (rather than directing a sequence of behaviors towards the male), we added 1 second to her association time with that male.

### Data Analysis

Statistical analyses were conducted using JMP 9.0 and 10.0 (SAS Institute, 2011, 2012) or R 2.15.1 and 3.0.1 [100]. We tested the distributions of dependent variables and employed GLMs or non-parametric statistics in cases where there were marked deviations from normality.

We used principal components analysis (PCA) to summarize female behaviors and interpreted the resulting principal components (PC) axes based on the published literature and on trials in which females made unambiguous choices. Some female behaviors we recorded are part of female rejection displays in other phrynosomatid lizards (e.g., lateral compression, push-ups, biting) [92], [94], [101], [102], whereas others have been used as indications of female preference (e.g., approach) [44]. However, the meaning of these female display behaviors has not been assessed in the *S. grammicus* complex specifically, and female sceloporines may perform similar behaviors in multiple contexts [74]. We assumed that copulations represented definitive choices of males by females, but the low number of copulations we observed during our experiment prevented us from using copulation alone as an index of female choice. Of the 34 total females in our experiment, 7 copulated with one of the males with whom they interacted. These interactions included 3 copulations with an SC sympatric male, 3 copulations with an SC allopatric male, and 1 copulation with a DC sympatric male. No females copulated with DC allopatric males. We used these unambiguous interactions to support our literature-based hypotheses regarding which behaviors indicate preference for or rejection of a male in *S. grammicus* from these two localities, in this particular context [45]. This technique allowed us to develop a female preference index based on the behaviors we observed most frequently.

**Figure 4:**
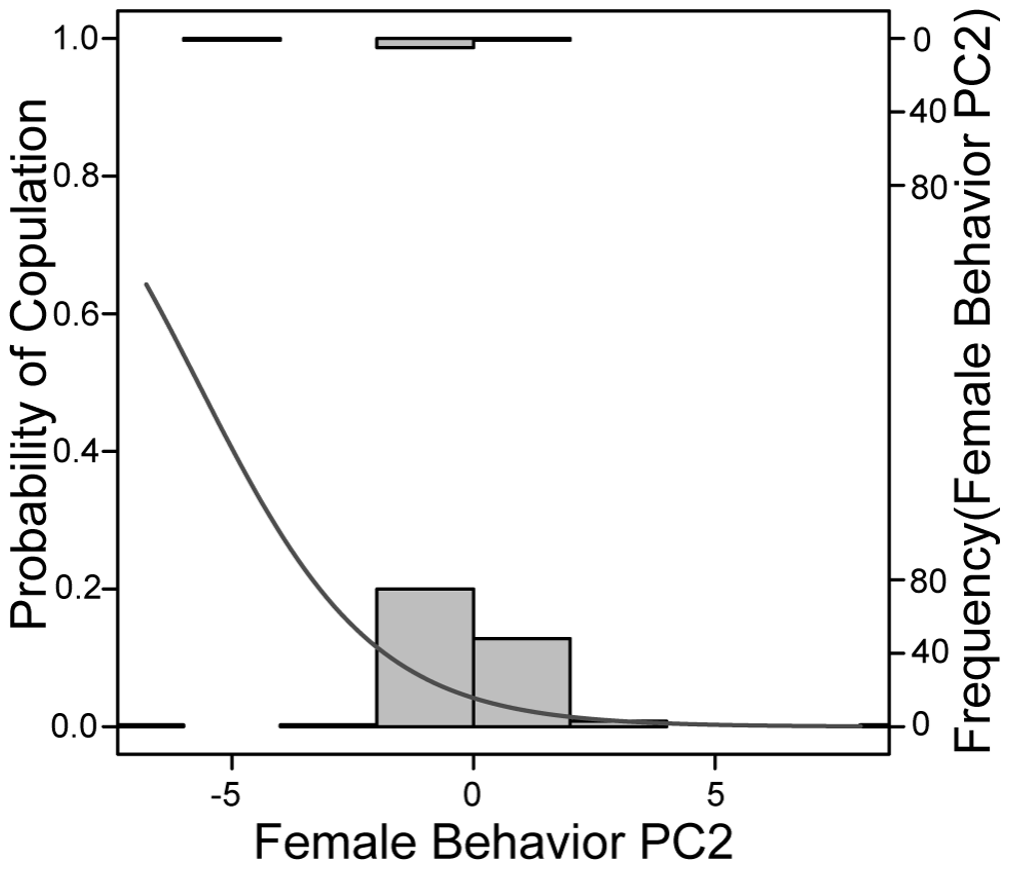
Logistic regression of copulation probability (0 = no, 1 = yes, left y-axis) vs. female behavior PC2 score (x-axis). The dark grey line shows the fitted logistic regression equation, while the histograms show how many male-female interactions fell into each bin of PC2 scores (right y-axis).

To summarize female behaviors, we first separated all trials into individual male-female interactions (i.e., two interactions per trial, or four total interactions per female) and ignored all information except which behaviors the female had performed and whether or not she had copulated with the male toward whom she performed those behaviors. We assigned interactions in which copulation occurred a score of 1 and interactions in which copulation did not occur a score of 0. We performed a principal components analysis (PCA) on a matrix of correlations between the incidences of all female behaviors except copulation [103], [104]. We saved the first three PC axes, because their eigenvalues were greater than 1 [105] (Table 2). We then performed generalized linear mixed model regression of copulation probability vs. these first three PC axes, including female ID as a random factor to avoid pseudoreplication. We performed the analysis with a binomial distribution and logit link, using the lmer function of the lme4 package in R 2.15.1 [100]. We removed non-significant effects in a stepwise procedure. Neither PC1 nor PC3 were significantly associated with copulation, but PC2 was significantly negatively associated with copulation probability (z_1,134_ = −2.54, P = 0.0111) (Fig. 4). Due to the negative relationship between PC2 and copulation probability, we considered PC2 to be an index of female rejection behavior (Fig. 4, Table 3). For details regarding the loadings of each behavior we scored on PC2, please see the Supplementary Material (Text S1).

**Table 2:** Loading matrix for PC axes 1–3 from a PCA on female behaviors other than copulation.

Behavior	PC1	PC2	PC3
Lateral Compression	0.719	0.426	0.370
Push-up	0.370	0.653	0.502
Bite	0.691	−0.529	0.015
Lick	0.809	−0.193	−0.100
Touch	0.725	−0.074	−0.277
Tail Wave	0.591	−0.223	−0.110
Tail Vibration	0.153	−0.415	0.641
Approach	0.688	0.349	−0.302
Retreat	0.710	−0.354	−0.032
Substrate Taste	0.448	0.521	−0.253
Eigenvalue	3.86	1.67	1.05
% Variation Explained	38.6%	16.7%	10.5%

**Table 3:** Composite variables used in this study, with the methods used to calculate them and explanations of why they were used.

Variable Name	Method of Calculation & Justification of Use
Rejection Index	PC axis number 2 from PCA on all female behaviors except copulation (Table 2).Calculated using each female’s interaction with each male(4 males per female) as unit of replication. Significantly negativelycorrelated with copulation probability in a logistic regression.
DiscriminationIndex	(Rejection Index towards Allopatric Male) - (Rejection Index towardsSympatric Male). Calculated separately for each trial, yielding twoDiscrimination Index scores per female. Measure of whether female exhibited morerejection behaviors towards the sympatric male than the allopatric male,within a single trial.
Discriminationdifferential	(Discrimination Index in DC Trial) - (Discrimination Index in SC Trial).Measure of whether a female’s rejection of the allopatric male (as measuredby the Discrimination Index) differed between DC and SC trials.

Based on the negative association between PC2 and copulation (Fig. 4) and also on the positive loadings (Table 2) of putative rejection behaviors such as lateral compression and push-ups [92], [94], [101], [102] on PC2, we used each female’s PC2 score towards each male with whom she interacted as a measure of her rejection of that male. Separately for each SC trial and each DC trial, we calculated a “discrimination index” to measure how much the female rejected the allopatric relative to the sympatric male (Table 3). We calculated the discrimination index by subtracting the female’s PC2 score towards the sympatric male from her PC2 score towards the allopatric male [44]. Finally, we subtracted each female’s discrimination index in her SC trial from her discrimination index in the DC trial to calculate a “discrimination differential” for each female (Table 3). This discrimination differential measured the degree to which a female directed more rejection behaviors toward the allopatric than the sympatric male in her DC trial relative to the degree to which she directed more rejection behaviors towards the allopatric than the sympatric male in her SC trial. A positive value of this discrimination differential would indicate that a female discriminated more between allopatric and sympatric males during her pure blue vs. pure white DC trial than during her SC trial (in which the males were more similar in coloration) (Fig. 3). If females can discriminate more easily between allopatric and sympatric males when the allopatric male is of an unfamiliar morph than when the allopatric male is of a familiar morph, we expect the discrimination differentials to be greater for females who participated in trial pairings with identical morph SC trials (Fig. 3) than for females who participated in trial pairings with dosage effect SC trials (Fig. 3). We used a general linear mixed model to examine the effect of SC trial type on female discrimination differential, while also assessing the effects of male display intensity (number of head bobs plus number of push-ups), female population, and male identity (as a random effect). We also conducted a similar analysis of whether female throat color morph influenced discrimination differential. Please see the Supplementary Material (Text S1) for further details on this analysis.

Our overall hypothesis in this study was that females would discriminate more between allopatric and sympatric males when the allopatric males had an unfamiliar throat color than when the allopatric males were of a familiar throat color morph. This hypothesis also predicts that females would reject allopatric males more strongly in dosage effect SC trials than in identical morph SC trials (Fig. 3). We tested this hypothesis by using a general linear model to examine the effect of SC trial type on female discrimination index within SC trials. Please see the Supplementary Material (Text S1) for further details about this analysis.

Because association time has been used as a measure of female preference in other work on mate choice [33], [37], [48], [102], we also conducted an analysis in which we compared the amount of time a female spent performing behaviors toward each male with our behavior-based rejection index (i.e., PC2) as a predictor of copulation. We concluded that our rejection index was a more appropriate measure of female behavior in this case, but for completeness, we conducted a parallel analysis using association time, rather than our PCA-based behavioral metric.

For comparability with our discrimination differential analysis, we first calculated the difference between the time a female spent with the allopatric male vs. the sympatric male within a trial (i.e., time with DC allopatric male - time with DC sympatric male and time with SC allopatric male - time with SC sympatric male). Then, we subtracted the time difference in the SC trial from the time difference in the DC trial. For brevity, we will refer to this measure as “association time differential.”

## Results

### Compliance with Assumptions of Statistical Tests

The distribution of the composite variable “discrimination differential” (Table 3) deviated significantly from normality (Shapiro-Wilk test, W = 0.92, P = 0.015). However, the skewness and kurtosis of the distribution (calculated using R 2.15.1 [100]) indicated the distribution was only slightly negatively skewed (skewness = −0.115) and moderately leptokurtic (kurtosis = 2.17). The variance was homogeneous with respect to the independent variable of most interest to our analysis (SC trial type; Bartlett’s test, P = 0.33). We thus relied on the relative robustness of ANOVA family models to minor violations of normality and performed our analysis using a general linear model approach with restricted maximum likelihood (REML).

### Effects of Male Identity on Discrimination Differential

Our sample size was not sufficient to allow us to include random effects of the identities of all four males with whom a female had interacted on her discrimination differential, while also considering all fixed effects of interest (female population, male display rates, and SC trial type). We therefore first fit a model including only our 4 random effects: SC sympatric male identity, SC allopatric male identity, DC sympatric male identity, and DC allopatric male identity (Table 4). Only DC sympatric male identity accounted for a variance component significantly different from zero (based on its 95% confidence interval, Table 4), so that was the only random effect we retained in subsequent tests.

**Table 4:** Components of variance in female discrimination differential explained by male identity (ID), estimated as random effects from a general linear model with REML.

Random Effect	# Males	Variance Ratio	Variance Component	Standard Error	95% CI Lower	95% CI Upper
SC Sympatric Male ID	29	−0.422	−1.59	2.31	−6.11	2.94
SC Allopatric Male ID	30	0.186	0.701	1.07	−1.39	2.80
DC Sympatric Male ID	28	1.42	5.34	2.46	0.529	10.2
DC Allopatric Male ID	28	−0.243	−0.916	1.05	−2.98	1.15
Residual			3.76	4.33	0.902	419
Total			7.30			

### Effects of SC Trial Type and Male Display Rates on Discrimination Differential

We initially fit a general linear model including DC sympatric male identity as a random effect and SC trial type, female population, SC sympatric male display intensity, SC allopatric male display intensity, DC sympatric male display intensity, DC allopatric male display intensity, and all 2-way interactions between SC trial type and the other fixed factors. We removed non-significant factors in a stepwise procedure, arriving at a final model that included SC trial type (F_2.041, 33_ = 370, P = 0.0024), SC allopatric male display intensity (F_2.037, 33_ = 38.0, P = 0.024), DC allopatric male display intensity (F_2.033, 33_ = 71.8, P = 0.013), and the interaction SC trial type by SC allopatric male display intensity (F_2.032, 33_ = 32.0, P = 0.029). The proportion of variance in discrimination differential among females that was explained by DC sympatric male identity continued to be significantly greater than zero (variance component = 7.82, 95% CI = 3.64, 12.0).

**Figure 5:**
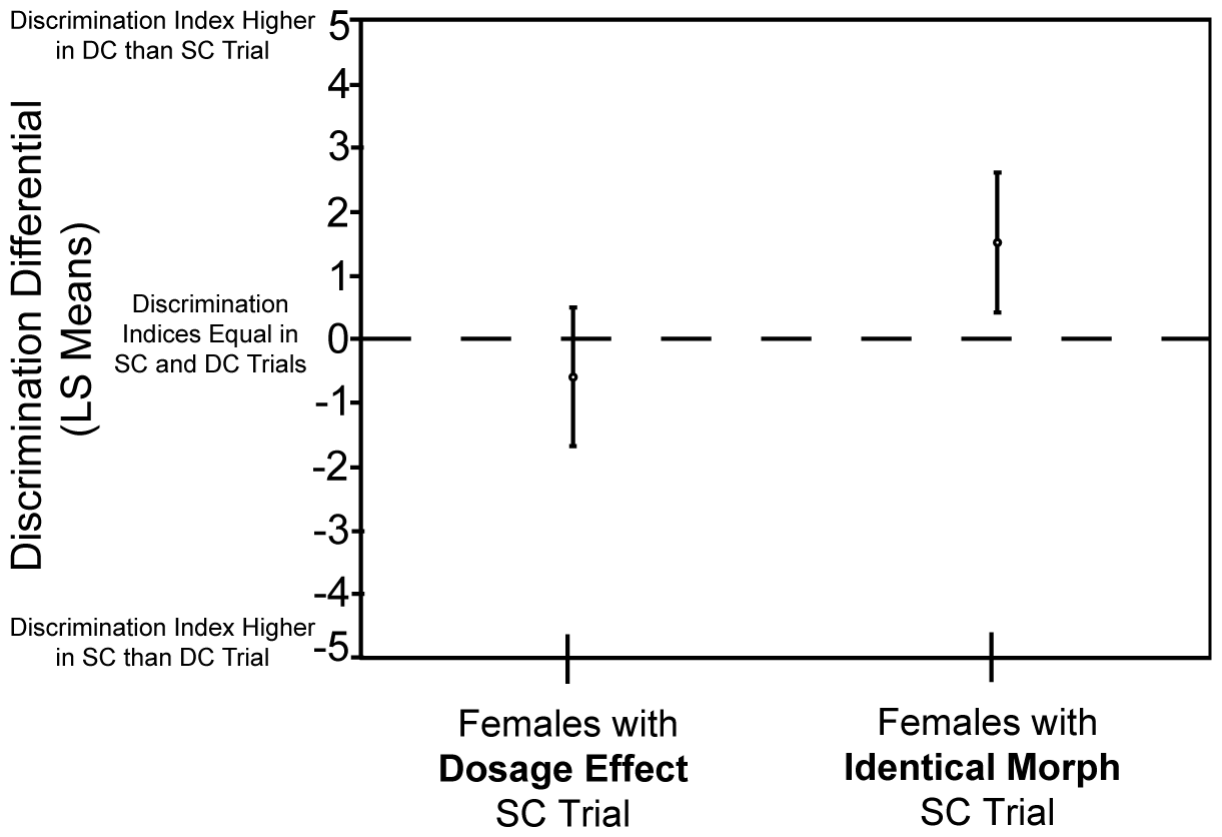
Least-squares (LS) mean discrimination differential was greater for females who participated in trial pairings with identical morph SC trials than for females in trial pairings with dosage effect SC trials. In addition, the LS mean discrimination differential was significantly greater than zero for females participating in trial pairings with identical morph SC trials but not for females participating in pairings with dosage effect SC trials. Circles represent LS means, while bars are ±95% confidence intervals. Dashed line represents a discrimination differential of zero.

The average discrimination differential was greater for females participating in trial pairings that included identical morph SC trials (Fig. 3) than for females participating in trial pairings that included dosage effect SC trials (Fig. 3, Fig. 5). Calculating the 95% confidence intervals (CI) of the least-squares (LS) means for each category of females revealed that the discrimination differential for females who experienced dosage effect SC trials was not significantly different from zero (LS mean = −0.609, 95% CI = −1.69, 0.477). The discrimination differential for females who experienced identical morph SC trials, however, was significantly greater than zero (LS mean = 1.50, 95% CI = 0.401, 2.60).

SC allopatric male display intensity had a negative effect on discrimination differential. This negative effect was stronger for trial pairings that included dosage effect SC trials than for pairings that included identical morph SC trials. That is, SC allopatric male display intensity had a stronger negative effect on discrimination differential for females who participated in dosage effect SC trials than for females who participated in identical morph SC trials. DC allopatric male display intensity had a positive effect on discrimination differential.

### Effects of Female Morph on Discrimination Differential and Comparison of SC Trial Types

In a parallel analysis to the one we conducted of the effect of SC trial type on discrimination differential, we found no effect of female morph on discrimination differential (F_4,8.108_ = 0.58, P = 0.68) (Fig. S1). We also compared discrimination indices (Table 3) between identical morph and dosage effect SC trials. Although the difference in discrimination indices was in the direction predicted by our hypothesis (i.e., greater for dosage effect trials than for identical morph trials), it was not close to significance (F_1,28.31_ = 0.25, P = 0.62) (Fig. S2). Please see the Supplementary Materials (Text S1) for further information about both these tests.

### Effects of SC Trial Type and Male Display Rates on Association Time Differential

Association time differential was normally distributed (Shapiro-Wilk test, W* = *0.97, P = 0.51). As with discrimination differential, we first performed a test including only random effects of the four male identities. The confidence intervals for all variance components overlapped zero, so we removed random effects from the model and fit a standard, fixed-effects linear model to investigate the possible effects of SC trial type and male display intensities.

We initially fit a model including female population, SC trial type, all four male display rates, and the interaction effects of all four male display rates with SC trial type on association time differential. We removed non-significant effects in a stepwise procedure, arriving at final model that included nearly significant effects of SC trial type (F_1,30_ = 3.5, P = 0.07) and SC allopatric male display rate (F_1, 30_ = 2.9, P = 0.10) and a significant effect of SC sympatric male display rate (F_1, 30_ = 5.4, P = 0.03). The association time differential was nearly significantly greater for females participating in identical morph SC trials than for females participating in dosage effect SC trials, but the 95% CI of association time differential for both types of females overlapped zero (Dosage Effect SC Trial: LS mean = −178 s, CI = −447 s, 90 s; Identical Morph SC Trial: LS mean = 370 s, CI = −161 s, 901 s). The effect of SC allopatric male display rate on association time differential was nearly significantly negative, and the effect of SC sympatric male display rate was significantly positive. For discussion of these results and further analyses supporting our decision to use our PCA-based behavioral metric as our primary measure of female response to males, please see the Supplementary Materials (Text S1).

## Discussion

Our findings suggest that female *S. grammicus* 1) respond to throat color when evaluating potential mates, 2) are capable of distinguishing between sympatric and allopatric males, and 3) differentiate between sympatric and allopatric males more strongly when allopatric males exhibit a throat color which is absent from a female’s home population. Previous research has revealed cases in which individuals mate assortatively based on sexual signals which are polymorphic within their population [35], [47] or based on sexual signals which differ between populations of the same species or between closely related species [25], [33], [36], [37], [74], [106]. Most of this research has focused on the possible role of morph losses in speciation [16], [40], or on the role of divergence in sexual signals in driving speciation [30], [107], [108]. To our knowledge, the *S. grammicus* complex currently represents the only known example of a taxon with polymorphic sexual signals in which some morphs (yellow and orange, in this case) occur in most or all populations, but other morphs are population-specific and never co-occur (blue vs. white in males) (Figs. 1 & 2, [65], [68]).

Our results indicate that females distinguished more between sympatric and allopatric males in pure blue vs. pure white trials than they did in trials where the two males were of the same color morph (i.e, yellow vs. yellow or yellow-orange vs. yellow-orange) (Fig. 3, Fig. 5). However, females did not distinguish more between sympatric and allopatric males in pure blue vs. pure white trials than in trials where the two males were of mixed morphs that differed by one color patch (i.e., blue-orange vs. white-orange or blue-yellow vs. white-yellow) (Fig. 3, Fig. 5). Other interpopulation mate choice trials have found that preferences can be asymmetric, with members of one population discriminating more strongly than members of the other population [74]. However, neither female population nor its interaction with SC trial type had significant effects on discrimination differential, indicating that the response of CPN females to white-throated males was similar to the response of SAA females to blue-throated males.

Few copulations occurred during our experiment, which was why we framed our analysis in terms of female rejection behaviors rather than female acceptance behaviors (Table 3). One male in each trial was from an allopatric population, so we would not, *a priori*, have necessarily expected females to accept those males as mates. The high incidence of rejection behaviors we observed is consistent with this hypothesis [31], [34], [36]. In addition, territory quality is an important influence on female mate choice in natural populations of many polygynous lizards [48], [90], [109], [110], so the fact that females could not evaluate male territories may have reduced female willingness to mate.

The large component of the variation in female discrimination differential explained by DC sympatric male identity occurred even though the number of unique males in this category was not substantially less than the number of unique males in the other three categories (Table 4). Our experiment did not assess female preference for traits other than throat color, nor evaluate female preference functions within populations. However, the effect of male identity for one experimental category, but not others, suggests female mate preference may be multimodal and context-dependent in *S. grammicus*, as it is in several other taxa, including some lizards [44], [46], [111]–[113]. Future research should investigate female preference functions for throat color and other traits within populations [34], [35], [45], [47], [90], [114] as well as possible variation in those functions among populations [74], [113], [115], [116].

The relationship between male display intensity (total number of head bobs plus total number of push-ups) and female discrimination differential may indicate that throat color, *per se*, is not the only signal to which females respond. The display intensities of sympatric males did not affect female discrimination differential for either trial category. However, the display intensities of allopatric males had significant effects in both SC and DC trials. SC allopatric male display intensity had a negative effect on female discrimination differential, indicating that females’ responses to SC and DC pairings were more similar when SC allopatric males displayed more. In contrast, the effect of DC allopatric male display intensity on discrimination differential was positive, indicating that females’ responses to DC and SC pairings differed more when DC allopatric males displayed more. Taken together, these two findings may suggest that, within trials, females distinguished more between sympatric and allopatric males when allopatric males displayed more. Allopatric males who displayed more may have exposed their throat coloration or other aspects of their courtship display more clearly to females, enhancing the females’ ability to identify their foreign origin [117], [118]. Head-bob and push-up displays vary within and among other species of *Sceloporus* [98], [99], [119], so it is possible that subtle variation exists between the courtship displays of males from SAA and CPN. Allopatric males who displayed more could therefore have been easier for females to differentiate from sympatric males, although our experiment did not explicitly test this hypothesis.

We chose CPN and SAA for our comparison of “blue male” and “white male” populations specifically because they are ecologically similar [75], [76]. Indeed, broader sampling of populations from northern and central Mexico has not revealed any obvious ecological pattern explaining why some populations of the *S. grammicus* complex exhibit the blue male morph, whereas other exhibit the white male morph [69]. The behavioral and sexual signal differences between CPN and SAA occur despite their ecological similarity and the apparently close evolutionary relationship between these two populations [50], [65], [79]. Our findings suggest that further studies of behavioral variation and female responses to sympatric vs. allopatric color morphs across other populations within the *S. grammicus* species complex are warranted. Such studies may help resolve both why the “blue vs. white” variation we have documented exists and what its current effects on gene flow within the species complex may be.

Some of the previously identified hybrid zones between chromosome races in the *S. grammicus* complex [51], [54]–[59] also represent contacts between populations with blue males and populations with white males, but this is not always the case [69]. While we did not perform karyotyping at CPN or SAA ourselves, the most recent data available [50] indicate that both populations most likely exhibit the 2N = 32 karyotype. It is therefore unlikely that these two populations represent the ends of a hybrid zone between two chromosome races, so we do not know whether crosses between them would exhibit the reduced hybrid fitness previously documented between populations of the *S. grammicus* complex differing in karyotype [59]. However, a recent study in *Uta stansburiana*, another species with throat color morphs that differ in reproductive tactics, found that male morphs varied in their reproductive compatibility across recently diverged populations with no difference in chromosome number [40]. That study did not assess whether females varied in their ability to differentiate allopatric and sympatric males of different morphs, and our study does not address what selective forces may have favored females’ ability to distinguish allopatric and sympatric males.

Our findings regarding the response of females to allopatric vs. sympatric male morphs, however, provide new insight into the mechanisms by which color polymorphism may drive speciation [14], [39]. Previous work [40] showed that postmating reproductive isolation could vary by morph in a similar system, but this study shows that premating reproductive isolation may also be morph-dependent in taxa where polymorphic sexual signals vary among populations. It is possible that similar patterns occur in other species with polymorphic sexual signals. Further sampling of multiple populations of such species may reveal ecological or phylogenetic patterns that account for variation in the number and frequency of morphs, and it may also clarify the processes by which polymorphic populations diverge during speciation [13]–[15], [39].

## Supporting Information

Figure S1 **Female discrimination differential (LS means, error bars show 95% CI) versus female throat color morph.** We found no effect of female color morph on discrimination differential.
(TIF)

Figure S2 **Female discrimination index (LS means, error bars show 95% CI) did not differ significantly between dosage effect and identical morph SC trials.**
(TIF)

Text S1 **Supplementary Materials and Methods, Results, and Discussion sections.**
(DOCX)

Data S1 **Data supplement, including female and male identities, throat color morphs, and behaviors performed during mate choice trials.**
(XLSX)
