# Supplementary figures and images for: Female Preference for Sympatric vs. Allopatric Male Throat Color Morphs in the Mesquite Lizard (Sceloporus grammicus) Species Complex
Source: PLoS One. 2014 Apr 9;9(4):e93197. doi: 10.1371/journal.pone.0093197 (PMC3981705; doi:10.1371/journal.pone.0093197)

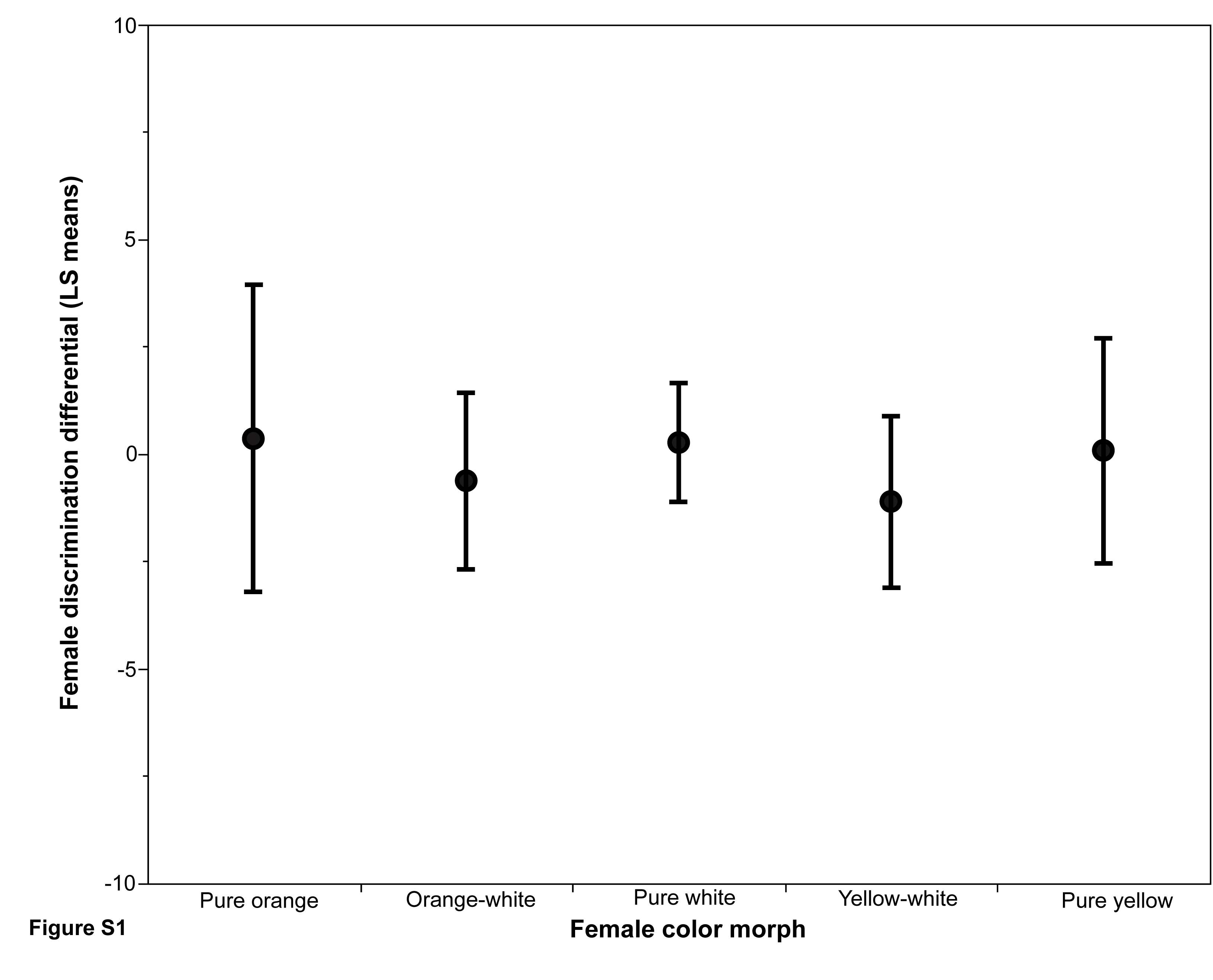

Supplement: Figure S1 — Female discrimination differential (LS means, error bars show 95% CI) versus female throat color morph. We found no effect of female color morph on discrimination differential. (TIF) [file pone.0093197.s001.tif]

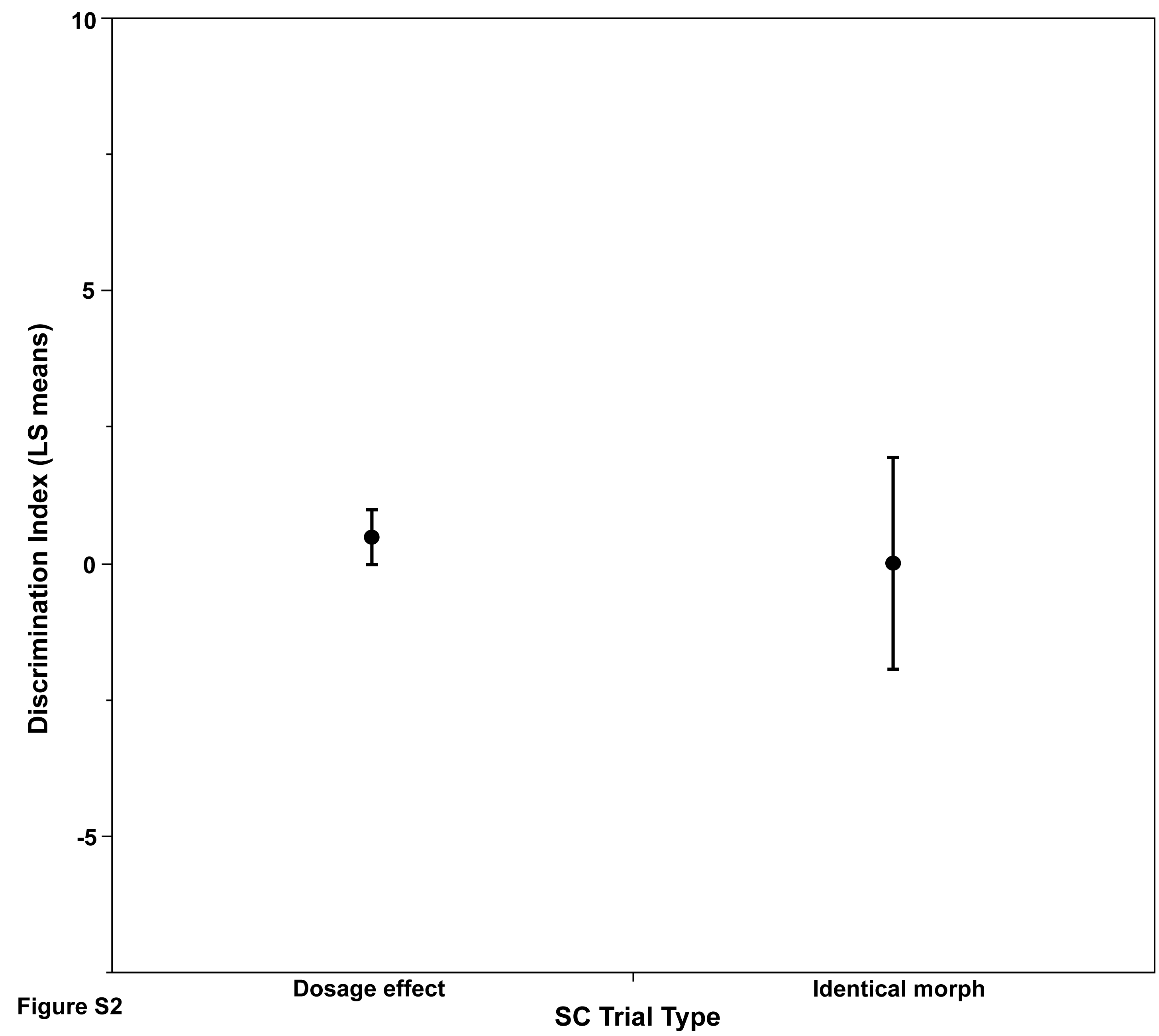

Supplement: Figure S2 — Female discrimination index (LS means, error bars show 95% CI) did not differ significantly between dosage effect and identical morph SC trials. (TIF) [file pone.0093197.s002.tif]
