# Supplementary material for: Female Preference for Sympatric vs. Allopatric Male Throat Color Morphs in the Mesquite Lizard (Sceloporus grammicus) Species Complex
Source: PLoS One. 2014 Apr 9;9(4):e93197. doi: 10.1371/journal.pone.0093197 (PMC3981705; doi:10.1371/journal.pone.0093197)
Supplement: Text S1 — Supplementary Materials and Methods, Results, and Discussion sections. (DOCX) [file pone.0093197.s003.docx]

**Supplementary Materials and Methods**

*Throat color scores:*

We classified males and females from each population into six morphs, three “pure” morphs and three “mixed” or “intermediate” morphs. At CPN, the pure male morphs included orange (Fig. 1A), blue (Fig. 1B), and yellow (Fig. 1C); while the intermediate male morphs included yellow-orange (Fig. 1D), blue-orange (Fig. 1E), and blue-yellow (Fig. 1F). In females, the pure morphs were orange (Fig. 1G), white (Fig. 1H), and yellow (Fig. 1I); while the intermediate morphs were yellow-orange (Fig. 1J), white-orange (Fig. 1K), and white-yellow (Fig. 1L).

At SAA, the pure male morphs included orange (Fig. 2A), white (Fig. 2B), and yellow (Fig. 2C); while the intermediate male morphs included yellow-orange (Fig. 2D), white-orange (Fig. 2E), and white-yellow (Fig. 2F). At SAA, females exhibited the same six morphs as males: the pure morphs were orange (Fig. 2G), white (Fig. 2H), and yellow (Fig. 2I); while the intermediate morphs were yellow-orange (Fig. 2J), white-orange (Fig. 2K), and white-yellow (Fig. 2L).

We assigned throat color scores immediately upon capturing a lizard. We also documented each individual’s throat color using digital photography. Before using males in trials, we visually compared them to ensure that males in identical morph SC trials were similar in color, males in dosage control SC trials exhibited two color patches, and males in DC trials were either pure blue or pure white, with no other color patches present on the throat. The same individual who scored lizards in the field (EB) also re-scored all males used in trials based on their photographs at least 1 year after the captures and trials. We used Cohen’s Kappa coefficient [1] to assess the repeatability of our scores, and the scores were highly repeatable (K = 0.90, P < 0.0001). We scored both male and female throat colors for the purposes of calculating population frequencies of the color morphs (Fig. 1M, Fig. 2M), but we used only male throat color scores as part of our experimental design.

*Female Behavior Analysis*

The behaviors with strong positive loadings on PC2, and thus a negative association with copulation, included lateral compression, push-ups, and substrate tastes (Table 1, Table 2). Lateral compression and push-ups (Table 1) have frequently been classified as rejection behaviors in female phrynosomatid lizards [2–5]. Substrate tasting appears to be a generalized exploratory behavior in *Sceloporus* lizards [6,7], and its positive loading on our rejection index may be explained by the fact that most females in our study rejected the males presented to them after approaching and evaluating them. Indeed, it is possible that females may benefit from obtaining chemosensory as well as visual information about males before accepting or rejecting them [8]. However, “approach” by females also loaded weakly positively on our rejection index, despite the fact that similar behaviors have been incorporated into metrics of female preference in other experiments using female phrynosomatid lizards [9,10]. While females approached males both before copulating with them and before performing rejection behaviors such as lateral compression or push-ups, the fact that rejection behaviors were more common in our experiments than copulation probably accounts for the positive loading of “approach” on our rejection index.

The behaviors tail vibration, bite, and retreat loaded strongly negatively on PC2 (Table 1, Table 2), implying that they were positively associated with copulation. Tail vibration has been classified as courtship behavior in male geckos [11] and observed as a response to predators in female *Podarcis sicula* [12]. While this behavior, like many lizard display behaviors, may appear in multiple contexts [13], our data suggest that tail vibration may be an indication of female acceptance of courtship in *S. grammicus.*

The negative loading of “bite” on our rejection index (Table 2) was more surprising, because this behavior is usually considered an unambiguous expression of rejection by females [3]. However biting by females occurred only a few times during our experiment, and its negative loading on PC2 probably resulted from a single trial in which a female first bit a male and retreated before eventually approaching and copulating with him (pers. obs. E. Bastiaans and M.J. Bastiaans). The association of “retreat” with copulation (Table 2) probably occurred because most females retreated from males soon after copulating with them or performing other behaviors weakly associated with copulation, such as “lick,” “touch,” or “tail wave” (Table 2). Our observations suggest that after females performed rejection displays such as lateral compression or push-ups, males usually ceased to court them, so those females were, counterintuitively, less likely to retreat than females who had not rejected a given male (pers. obs. E. Bastiaans and M.J. Bastiaans).

Because association time is often used to measure mate preference [5,14–16], we conducted a parallel analysis to the one using discrimination differential, with association time substituted for our rejection index (please see main text Materials and Methods and Results sections). However, studies of mate choice in other taxa have found that measuring approach behavior or association time can fail to differentiate between acceptance behavior and rejection or aggressive behavior [17,18]. We were therefore uncertain how association time should be interpreted in our particular context. We performed several analyses in R 3.0.1 [19] to compare association time with our behavior-based metric as measures of female preference or rejection. We defined association time as the time each female had spent performing behaviors toward each of the four males with which she interacted. We used R 3.0.1 [19] to assess whether association time predicted female copulation as well or better than the second axis from our principal components analysis of female behaviors (female behavior PC2). We also checked whether the correlation between female behavior PC2 and association time was the same for different types of female interactions. This analysis was intended to give us insight into whether time a female spent with a sympatric male could be interpreted the same way as time a female spent with an allopatric male. We analyzed SC and DC trials separately and regressed association time on female behavior PC2, testing for an interaction with male type.

*Test for effects of female morph on discrimination differential*

We chose females for the trials randomly from among the females we had captured, following the approach described in [20]. The females from SAA we used included 1 pure orange, 3 pure yellow, 8 pure white, 3 white-orange, and 2 white-yellow females. The females from CPN we used included 1 pure orange, 7 pure white, 4 white-orange, and 5 white-yellow females. These proportions are relatively similar to the proportions of females of each morph present in the population as a whole (Fig. 1, Fig. 2). To determine whether our measure of female discrimination against allopatric males in DC vs. SC trials (discrimination differential, Table 3) differed among female morphs, we performed a parallel analysis to the one in which we found effects of SC trial type and allopatric male display rates (please see main Results section). We used JMP 10 (SAS Intitute, 2012) to test whether female morph affected discrimination differential, using a general linear mixed model with DC sympatric male ID as a random factor.

*Comparison of discrimination differential between identical morph and dosage effect SC trials*

We structured our main analysis using individual females as our unit of analysis in order to account for the considerable variation among females in behavior and improve our statistical power. Pairing each DC trial with an SC trial also allowed us to separate a female’s response to a novel morph, *per se*, from her response to an allopatric male. Our hypothesis predicted that females would exhibit stronger courtship rejection towards allopatric males in DC trials than in SC trials. Along the same lines, we might predict that females participating in dosage effect SC trials would exhibit stronger rejection of allopatric males than females participating in identical morph SC trials (Fig. 3). We tested for this possibility by comparing the discrimination indices (Table 3) between females who participated in identical morph vs. dosage effect SC trials (Fig. 3). We used JMP 10 (SAS Institute 2012) to fit a general linear mixed model testing the effect of both males’ identities and several fixed factors on female discrimination index (Table 3) in the two categories of SC trials we performed (Fig. 3).

**Supplementary Results**

*Comparison of discrimination differential and association time*

Female behavior PC2 was negatively associated with copulation probability (mixed-model logistic regression using the lme4 package in R 3.0.1 [19], with PC2 as a fixed factor and female ID as a random factor; z = -2.54, P = 0.011) (please also see main text). When we performed an identical analysis using association time rather than female behavior PC2, association time was not quite significantly associated with female copulation, although the trend was positive (z = 1.88, P = 0.06).

To perform our analyses of the relationship between female behavior PC2 and association time for different types of female-male interactions, we used the lme4 package in R 3.0.1 to fit a generalized linear mixed model regression of association time on female behavior PC2, with male type (sympatric or allopatric) as a fixed factor and female ID as a random factor. We used a Poisson distribution and log link function. We included the interaction effect of male type with female behavior PC2, to determine whether the relationship between PC2 and association time differed, depending on whether a female was associating with a sympatric male or an allopatric male. For SC trials, there were significant effects of PC2 (z = 32.6, P < 2 x 10^-16^), male type (z = 5.3, P = 1.5 x 10^-7^), and the interaction PC2 by male type (z = -9.2, P < 2 x 10^-16^). For DC trials, there were significant effects of PC2 (z = 14.9, P < 2 x 10^-16^), male type (z = 23.4, P < 2 x 10^-16^), and the interaction PC2 by male type (z = -39.4, P < 2 x 10^-16^). That is, the particular behaviors performed during the time a female associated with a male appear to have differed depending on whether she was associating with a sympatric male or with an allopatric male.

*Does female morph affect discrimination differential?*

We used a general linear mixed model with discrimination differential as the response variable to test for an effect of female color morph. As in our analysis of the effect of SC trial type, we included DC sympatric male ID as a random factor. We initially fit a general linear model including female morph, female population, SC sympatric male display intensity, SC allopatric male display intensity, DC sympatric male display intensity, and DC allopatric male display intensity as fixed factors. We removed non-significant factors in a stepwise procedure, until female morph was the only fixed factor remaining. We found no effect of female morph on discrimination differential (F_4,8.108_ = 0.58, P = 0.68) (Fig. S1).

*Do discrimination indices differ between identical morph and dosage effect SC trials?*

We initially fit a model including SC sympatric male identity and SC allopatric male identity as random effects and SC trial type, female population, SC sympatric male display rate, and SC allopatric male display rate as fixed effects. We also included the two-way interactions between SC trial type and the two males’ display rates. We removed non-significant fixed effects in a stepwise procedure, but none of the factors we included were ever significant in any model (all P > 0.05). In particular, although the difference in discrimination indices between females participating in identical morph SC trials and females participating in dosage effect SC trials was in the direction predicted by our hypothesis (i.e., greater for dosage effect trials than for identical morph trials), it was not close to significance (F­_1,28.31_ = 0.25, P = 0.62) (Fig. S2).

**Supplementary Discussion**

Our analysis of association time differential yielded results that, although not quite significant, were qualitatively similar to the results of our analysis of discrimination differential. In both cases, females differed more in their responses to allopatric vs. sympatric males when those males were more different from one another. That is, the difference between DC and SC trials was greater for females who experienced identical morph SC trials than for females who experienced dosage control SC trials (Fig. 5, see also main text Results section). Also, in both cases, increased display by SC allopatric males was correlated with decreased differences between female responses in the two trials.

If association time measures predominantly rejection behaviors, we can interpret the results of our association time differential analysis in the same way as we did the results of our discrimination differential analysis (please see main text Discussion). However, association time is often interpreted as a measure of mate preference, not rejection [5,14–16]. If we interpret association time this way, our results suggest that females preferred allopatric males over sympatric males nearly significantly more strongly when the allopatric male was more different in color from the sympatric male. This result would be novel and surprising, but it is not consistent with personal observations (E. Bastiaans and M.J. Bastiaans) of female behavior during trials, or with the low number of copulations we observed. We also suspect that some of the time females spent in males’ display areas was motivated by thermal requirements, rather than mate preference. To encourage females to notice and interact with males, we placed heat lamps over both male display areas but not over the area of the trial chamber in which we initially placed the female. An alternative explanation for why a female spent time near a given male might therefore be that she was seeking basking opportunities, rather than seeking to mate. We argue that, in this context, a female’s display behaviors are a better measure of her preference for or rejection of a given male than association time.

Our analysis of the relationship between PC2 (our measure of female mate rejection) and association time also revealed significant interaction effects of PC2 by male type on association time for both SC and DC trials. This indicates that the relationship between association time and PC2 was not the same for all male types with which each female interacted. Based on these results, we also argue that the time a female spent with a sympatric male cannot necessarily be interpreted in the same way as the time she spent with an allopatric male, making association time even more difficult to interpret as a measure of female preference in this case. However, the differences between the behaviors represented by female association time with different categories of males may help to explain another puzzling result: the positive effect of SC sympatric male display rate on association time differential. If association time with SC sympatric males does represent preference, and females preferred males who displayed more, this effect could have decreased the difference in association time between sympatric and allopatric males in SC trials, thus tending to increase the difference between SC and DC trials.

Although further work in this area is needed, our results here seem to suggest that, although association time may be an appropriate method for scoring mate choice in some cases [21], it may sometimes represent motivations other than mating [17,18]. In many situations, it may also be valuable to explore other measures of mate preference or rejection, particularly in taxa that exhibit stereotyped rejection and courtship behaviors that can be reliably scored and related to copulation probability [10,17].

**Literature Cited - Supplementary Material**

1. Cohen J (1960) A Coefficient of Agreement for Nominal Scales. Educ Psychol Meas 20: 37–46. doi:10.1177/001316446002000104.

2. Cooper Jr. WE (1984) Female secondary sexual coloration and sex recognition in the keeled earless lizard, *Holbrookia propinqua*. Anim Behav 32: 1142–1150. doi:10.1016/S0003-3472(84)80230-4.

3. Cooper WE (1988) Aggressive Behavior and Courtship Rejection in Brightly and Plainly Colored Female Keeled Earless Lizards (*Holbrookia propinqua*). Ethology 77: 265–278. doi:10.1111/j.1439-0310.1988.tb00210.x.

4. Hews DK, Castellano M, Hara E (2004) Aggression in females is also lateralized: left-eye bias during aggressive courtship rejection in lizards. Anim Behav 68: 1201–1207. doi:10.1016/j.anbehav.2003.11.024.

5. Swierk L, Myers A, Langkilde T (2013) Male mate preference is influenced by both female behaviour and morphology. Anim Behav 85: 1451–1457. doi:10.1016/j.anbehav.2013.03.042.

6. Duvall D (1979) Western fence lizard (*Sceloporus occidentalis*) chemical signals. I. Conspecific discriminations and release of a species-typical visual display. J Exp Zool 210: 321–325. doi:10.1002/jez.1402100215.

7. Quinn VS, Hews DK (2000) Signals and behavioural responses are not coupled in males: aggression affected by replacement of an evolutionarily lost colour signal. Proc R Soc B Biol Sci 267: 755–758.

8. Martín J, López P (2006) Links between male quality, male chemical signals, and female mate choice in Iberian Rock Lizards. Funct Ecol 20: 1087–1096. doi:10.1111/j.1365-2435.2006.01183.x.

9. Hamilton PS, Sullivan BK (2005) Female mate attraction in ornate tree lizards, *Urosaurus ornatus*: a multivariate analysis. Anim Behav 69: 219–224. doi:10.1016/j.anbehav.2004.03.011.

10. Bleay C, Sinervo B (2007) Discrete genetic variation in mate choice and a condition-dependent preference function in the side-blotched lizard: implications for the formation and maintenance of coadapted gene complexes. Behav Ecol 18: 304–310. doi:10.1093/beheco/arl101.

11. Steele LJ, Cooper WE (1997) Investigations of Pheromonal Discrimination between Conspecific Individuals by Male and Female Leopard Geckos (*Eublepharis macularius*). Herpetologica 53: 475–484. doi:10.2307/3893261.

12. Downes SJ, Bauwens D (2002) Does reproductive state affect a lizard’s behavior toward predator chemical cues? Behav Ecol Sociobiol 52: 444–450. doi:10.1007/s00265-002-0538-3.

13. Hardwick KM, Robertson, Jeanne M., Rosenblum EB (2013) Asymmetrical mate preference in recently adapted white sands and black lava populations of *Sceloporus undulatus*. Curr Zool 59.

14. Swierk L, Ridgway M, Langkilde T (2012) Female lizards discriminate between potential reproductive partners using multiple male traits when territory cues are absent. Behav Ecol Sociobiol 66: 1033–1043. doi:10.1007/s00265-012-1351-2.

15. Summers K, Symula R, Clough M, Cronin T (1999) Visual mate choice in poison frogs. Proc R Soc Lond B Biol Sci 266: 2141–2145. doi:10.1098/rspb.1999.0900.

16. Reynolds RG, Fitzpatrick BM (2007) Assortative Mating in Poison-Dart Frogs Based on an Ecologically Important Trait. Evolution 61: 2253–2259. doi:10.1111/j.1558-5646.2007.00174.x.

17. Egger B, Obermüller B, Eigner E, Sturmbauer C, Sefc KM (2008) Assortative mating preferences between colour morphs of the endemic Lake Tanganyika cichlid genus *Tropheus*. Hydrobiologia 615: 37–48.

18. Zoppoth P, Koblmüller S, Sefc KM (2013) Male courtship preferences demonstrate discrimination against allopatric colour morphs in a cichlid fish. J Evol Biol 26: 577–586. doi:10.1111/jeb.12074.

19. R. Core Development Team (2012) R: a language and environment for statistical computing. Vienna, Austria: R Foundation for Statistical Computing.

20. Lancaster LT, Hipsley CA, Sinervo B (2009) Female Choice for Optimal Combinations of Multiple Male Display Traits Increases Offspring Survival. Behav Ecol 20: 993–999. doi:10.1093/beheco/arp088.

21. Jeswiet SB, Godin J-GJ (2011) Validation of a Method for Quantifying Male Mating Preferences in the Guppy (*Poecilia reticulata*). Ethology 117: 422–429. doi:10.1111/j.1439-0310.2011.01891.x.
